# A fundamental study revisited: Quantitative evidence for territory quality in oystercatchers (*Haematopus ostralegus*) using GPS data loggers

**DOI:** 10.1002/ece3.2581

**Published:** 2016-12-20

**Authors:** Philipp Schwemmer, Stefan Weiel, Stefan Garthe

**Affiliations:** ^1^ Research & Technology Centre (FTZ)University of KielBüsumGermany

**Keywords:** *Ensis directus*, fitness, flight distance, foraging behavior, Germany, nocturnal foraging, predation, Wadden Sea

## Abstract

A fundamental study by Ens et al. (1992, *Journal of Animal Ecology*, 61, 703) developed the concept of two different nest‐territory qualities in Eurasian oystercatchers (*Haematopus ostralegus*, L.), resulting in different reproductive successes. “Resident” oystercatchers use breeding territories close to the high‐tide line and occupy adjacent foraging territories on mudflats. “Leapfrog” oystercatchers breed further away from their foraging territories. In accordance with this concept, we hypothesized that both foraging trip duration and trip distance from the high‐tide line to the foraging territory would be linearly related to distance between the nest site and the high tide line. We also expected tidal stage and time of day to affect this relationship.
The former study used visual observations of marked oystercatchers, which could not be permanently tracked. This concept model can now be tested using miniaturized GPS devices able to record data at high temporal and spatial resolutions.
Twenty‐nine oystercatchers from two study sites were equipped with GPS devices during the incubation periods (however, not during chick rearing) over 3 years, providing data for 548 foraging trips. Trip distances from the high‐tide line were related to distance between the nest and high‐tide line. Tidal stage and time of day were included in a mixing model.
Foraging trip distance, but not duration (which was likely more impacted by intake rate), increased with increasing distance between the nest and high‐tide line. There was a site‐specific effect of tidal stage on both trip parameters. Foraging trip duration, but not distance, was significantly longer during the hours of darkness.
Our findings support and additionally quantify the previously developed concept. Furthermore, rather than separating breeding territory quality into two discrete classes, this classification should be extended by the linear relationship between nest‐site and foraging location. Finally, oystercatcher′s foraging territories overlapped strongly in areas of high food abundance.

## 1 Introduction

**Figure 1:**
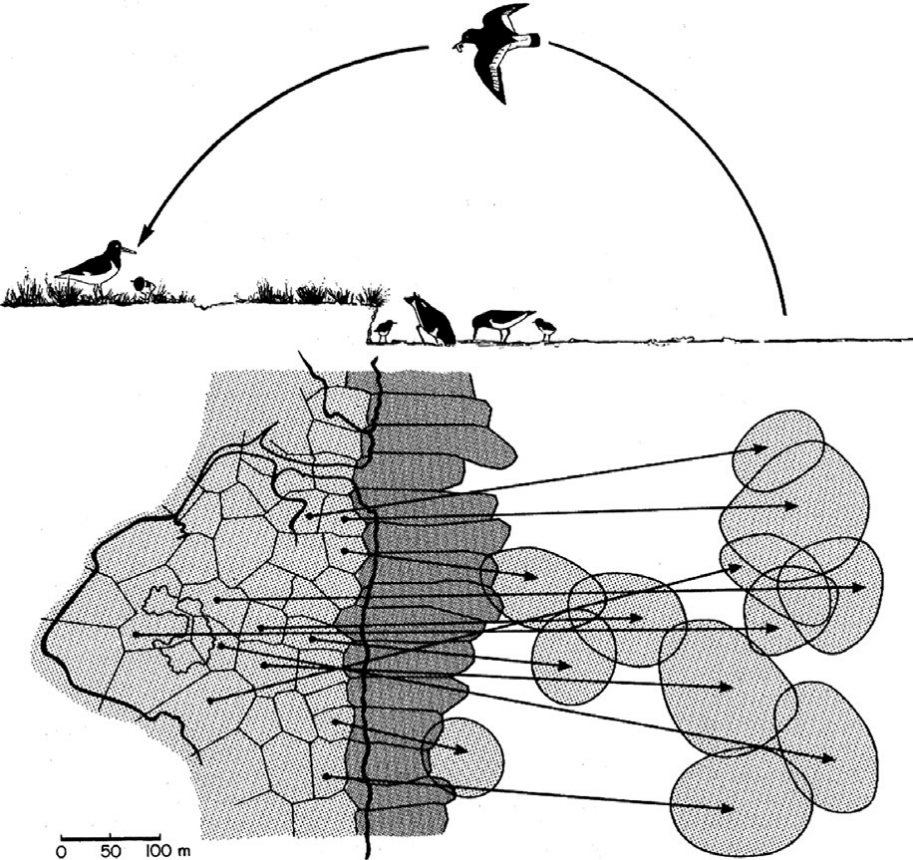
Concept of territory use in oystercatchers taken from Ens et al. (1992). “Resident” oystercatchers occupy breeding territories next to the high‐tide line (bold black line) and use foraging territories with direct access to the tidal flats (dark shaded areas), whereas “leapfrog” oystercatchers occupy breeding territories further inland and need to cross foraging territories of residents to reach their own foraging territories (light shaded areas)

Optimal food provision for chicks is essential for maintaining the fitness of adult and juvenile birds (e.g., Schwagmeyer & Mock, 2008; Shoji et al., 2015; van Oers, Kohn, Hinde, & Naguib, 2015; Whiteside, Sage, & Madden, 2015). The distance between profitable foraging grounds and nest territories is an important factor in food provisioning (Boersma & Rebstock, 2009; Brzorad, Maccarone, & Stone, 2015; Hull et al., 2001; Paiva et al., 2015). Accordingly, the location of the breeding territory with respect to distance from the intertidal foraging grounds was shown to be crucial for high offspring survival in Eurasian oystercatchers (*Haematopus ostralegus*, L.) (Ens, Kersten, Brenninkmeijer, & Hulscher, 1992; van de Pol, Bruinzeel, Heg, van der Jeugd, & Verhulst, 2006b). Ens et al. (1992) suggested that oystercatchers inhabit two types of breeding territories of different qualities. “Resident” oystercatchers have nest sites close to the high‐tide line, and given that their foraging territory is located directly adjacent to the intertidal mudflats (Figure 1), they only need to spend a short time commuting between nesting and foraging areas. In contrast, “leapfrog” oystercatchers breed further inland and occupy foraging territories further away from the high‐tide line (Figure 1). These birds therefore need to spend longer flying to reach their foraging territories, “leapfrogging” the residents’ foraging territories. This results in leapfrogs having lower reproductive success, because of the reduced time available to search for prey and feed their chicks (Ens et al., 1992; van de Pol, Bakker, Saaltink, & Verhulst, 2006a). The lower quality of leapfrog breeding territories is reflected by a lower social dominance of individuals inhabiting those territories (Bruinzeel, van de Pol, & Trierweiler, 2006; Ens, van de Pol, & Goss‐Custard, 2014; Heg, Ens, van der Jegd, & Bruinzeel, 2000), as well as lower survival rates and fitness of offspring (van de Pol et al., 2006a, b). Breeding territories furthest from the high‐tide line should thus be of the lowest quality, because oystercatchers from these nest sites need to cross the breeding territories that lie between their own breeding territory and the high‐tide line, as well as the foraging territories of at least some resident oystercatchers, to reach their own foraging territories.

This concept developed by Ens et al. (1992) was based on visual observations of marked individuals. It was therefore not possible to quantify the exact distances travelled by individual oystercatchers while commuting between foraging and breeding territories, to observe individual oystercatchers far away from the observer, or to observe their behavior at night.

Miniaturized GPS data loggers are now available that enable us to record the areas utilized by species within the size range of oystercatchers with high spatial and temporal resolutions (e.g., Schwemmer & Garthe, 2011; Schwemmer, Güpner, Adler, Klingbeil, & Garthe, 2016; Shamoun‐Baranes et al., 2012). Employing these devices thus enables the distance travelled by an individual oystercatcher between its nest site and foraging territory, as well as the time spent away from its nest site, to be quantified precisely.

We therefore revisited and extended the concept of Ens et al. (1992) by exploring the following hypotheses. (1) Distance travelled from the high‐tide line to the foraging territory would be positively correlated with the distance between the nest site and the high‐tide line. Ens et al.'s (1992) concept only classified breeding territories as low (i.e., leapfrogs) or high quality (i.e., residents). If the social dominance of oystercatchers breeding furthest from the high‐tide line was indeed lowest (Ens et al., 2014), they would be excluded from nearby foraging territories and forced to travel the longest distance to their intertidal foraging territories, resulting into a linear correlation between distance travelled from the high‐tide line and distance between the nest site and high‐tide line. (2) Similarly, the duration of foraging trips (i.e., time spent away from breeding territory) should increase with increasing distance between the nest site and the high‐tide line, because the birds would spend longer commuting between their nest and foraging sites. (3) Tidal stage would also be expected to impact on these relationships, because foraging territories further down the shore will be submerged earlier than those close to the high‐tide line. (4) Finally, foraging trips made during darkness should generally take longer, because the birds would spend more time searching for prey using tactile means (Schwemmer & Garthe, 2011; Sitters, 2000; Sutherland, 1982).

We tested these hypotheses to quantify the concept presented by Ens et al. (1992), using a dataset recorded by GPS data loggers over three different years from two different sites in the eastern Wadden Sea.

## 2 Materials and Methods

### 2.1 Study area

**Figure 2:**
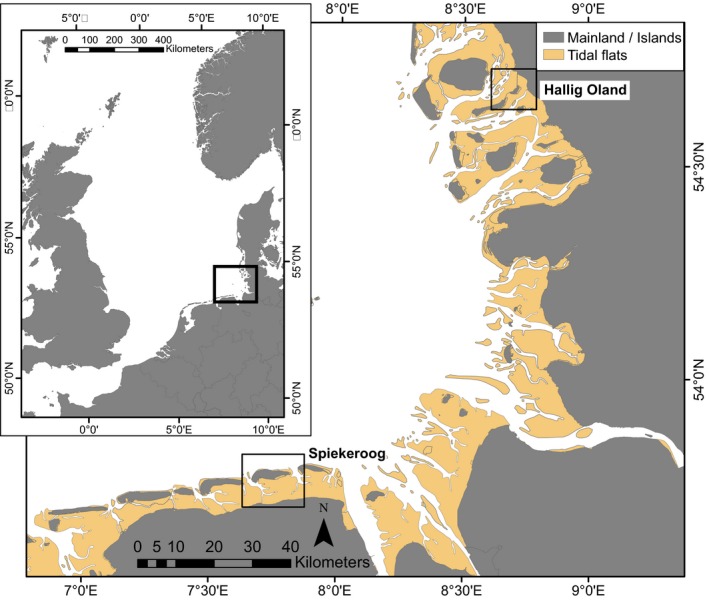
Location of the study area within Europe (small inserted map), and location of the study sites Hallig Oland (northern rectangle on large map) and Spiekeroog (southern rectangle on large map)

The study was performed on two different islands in the northeastern (Hallig Oland; 54°40′39″N, 8°42′14″E) and southeastern (Spiekeroog; 53°46′19″N, 7°41′49″E) Wadden Sea. Hallig Oland is a marsh island, completely surrounded by intertidal mudflats during low tide. Except of the northwestern part the island is surrounded by salt marshes. Two major tidal creek systems are running parallel to the island in the north and south. Oystercatchers are breeding both, in the salt marsh as well as in the meadows. Spiekeroog is a dune island with salt marshes and adjacent intertidal mudflats only in the south. Oystercatchers are breeding both in the salt marsh as well as in dunes. A major tidal creek system is entering the mudflats along the western tip of the island from north to south (Figure 2). For a more detailed description of the study sites, see Schwemmer and Garthe (2011) and Schwemmer et al. (2016). There were about 350 breeding pairs of oystercatchers on Oland, and about 660 breeding pairs on Spiekeroog during the study period. Oystercatchers on both islands bred at sites close to the high‐tide line (residents), as well as at sites further from the high‐tide line (leapfrogs) (Ens et al., 1992).

### 2.2 Data collection

A total of 29 oystercatchers were caught using walk‐in nest traps during incubation from May 15 to June 6 over a three year period, from 2008 to 2010 (Table 1). We were only able to collect GPS data during the incubation period because it was necessary to catch the birds back on the nests to take off the devices. In contrast, Ens et al. (1992) performed visual observations during the whole breeding period. The geographical positions of the nests were recorded using handheld GPS devices (76CSx, Garmin, Garching). Birds were measured, weighed, ringed, and dyed with livestock‐marking paint to facilitate identification in the field, and a GPS data logger (Mini‐GPS; Earth & Ocean Technologies, Kiel, Germany) was attached to the tail feathers using adhesive TESA tape (Wilson et al., 1997). The devices employed in 2008 weighed 18 g, while those employed in consecutive years were an updated version (Mini‐GPS 2) with an improved battery lifetime (enabling a higher amount of recorded trips during the equipment period), weighing 14 g. Oystercatchers showed a mean body mass of 523.3 g (± 47.2 g). Thus, the devices accounted for a mean of 3.4% (2008) and 2.7% (following years), respectively, of the body mass of the oystercatchers. This device mass is in range with the suggestion given by Phillips, Xavier, and Croxall (2003) to avoid device effects. It has previously been shown that these devices are unlikely to affect the foraging and breeding behaviors of the birds (Schwemmer & Garthe, 2011). The birds were released immediately after attachment of the devices and quickly returned to their nest territories.

**Table 1:** Numbers of equipped oystercatchers and foraging trips recorded at the two study sites over a three year period

Year	Hallig Oland		Spiekeroog		Total
	2008	2009	2009	2010	
Number of equipped birds	8	7	7	7	29
Number of trips	61^a^	116	146	225	548

a Number of trips was lower during 2008 as GPS devices had a limited battery lifetime and were improved in consecutive years.

The GPS devices recorded geographical position (inaccuracy 2–20 m) at intervals of 2 min plus an additional period of 6–22 sec for the satellite uplink, time of day, and velocity. The devices recorded data for 3–10 days, depending on battery power and uplink time. The birds were recaptured after a maximum of 13 days and the devices were removed.

### 2.3 Data analysis and statistics

The data were downloaded and visualized using geographical information system (GIS) software (ArcGIS 10.1; ESRI, 2011). The start of a foraging trip from the breeding territory to the mudflat could be recognized by an increase in ground speed following a change in geographical position. A trip comprised of the last position in the breeding territory, the movement to the mudflat, the foraging time on the mudflat, and the return movement until the first position within the breeding territory. Between four and 61 foraging trips per individual bird could be identified. The numbers of foraging trips per year and per study site are shown in Table 1. We computed the overall foraging trip duration (i.e., time from last position recorded in the breeding territory or on the nest before starting foraging trip until first position in the breeding territory or on the nest after the return trip, in minutes) and the maximum foraging trip distance (i.e., furthest distance of the foraging trip from the high‐tide line, in meters). These two parameters were used as dependent variables in the statistical model (see below).

The following five parameters were used as predictors. (1) Nest distance: The distance in meters from each oystercatcher nest to the high‐tide line was computed using GIS (mean nest distance: 124 m, minimum: 15 m, maximum: 315 m). (2) Time until high tide: The time (in minutes) to the next or previous high tide (depending on which high tide was closer) was calculated using tidal charts, after half the foraging trip had passed. (3) Time class: Time of day was classified as “day” (i.e., all daylight hours, including the time of civil twilight) or “night” (i.e., hours of darkness), according to Schwemmer and Garthe (2011). (4) Study site, and (5) study year were included as additional predictors. The numerical predictors (i.e., nest distance and time until high tide) were scaled and centered to enable the comparison of model effect sizes.

We applied generalized linear mixed effect models (Bolker et al., 2008; Faraway, 2006; Venables & Ripley, 2002) using the package lme4 in R (Bates & Maechler, 2009). Trip duration and maximum trip distance were used as dependent variables with study site, year, time of day, and nest distance as predictors. For each model, the individual bird identification number was used as a random factor to correct for pseudoreplication caused using multiple observations of the same individual. We checked the model for unequal variance structures (heteroscedasticity) by plotting standardized residuals against fitted values and looked at the qq‐plots of the residuals and random effects to check for normality of errors and transformed the response variables if necessary. Model selection was based on maximum likelihood ratio tests. For tests of main effects, interaction terms were removed from the model. Nonsignificant interactions between predictors were also removed from the model. Main effects were kept in the model (even if they were nonsignificant) to avoid overfitting of the models (Whittingham, Stephens, Bradbury, & Freckleton, 2006). To obtain the posterior distribution, we directly simulated 10,000 values from the joint posterior distribution of the model parameters using the function sim of the package arm (Gelman & Hill, 2007). The means of the simulated values from the joint posterior distributions of the model parameters were used as estimates, and the 2.5% and 97.5% quantiles as lower and upper limits of the 95% credible intervals. Posterior distributions of fitted values were obtained by calculating 10,000 fitted values each with a different set of model parameters from the posterior distribution. Again, the mean and the 2.5% and 97.5% quantiles of these 10,000 values were used as the estimate with a 95% credible interval.

All analyses were carried out using the free software package R3.2.2 (R Development Core Team, 2015).

## 3 Results

**Figure 3:**
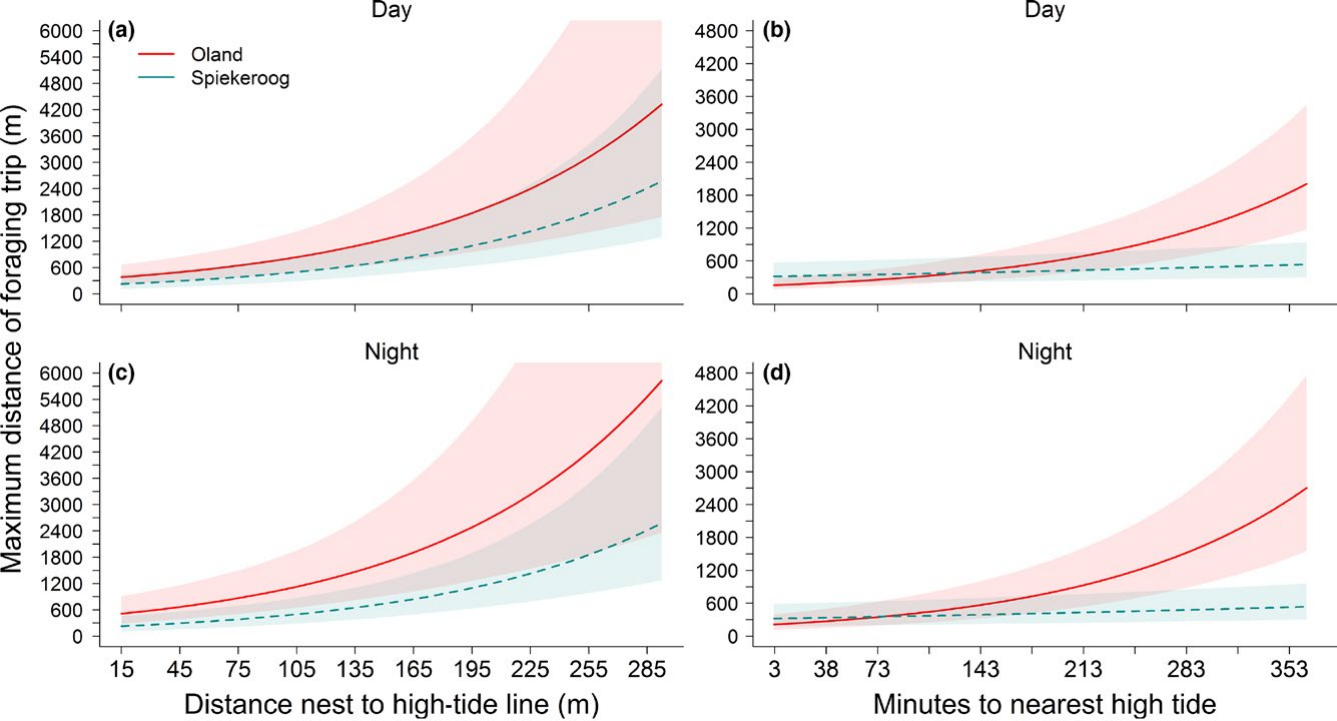
Model predictions for foraging trip distances in relation to distance between nest and high‐tide line during (a) day and (c) night, as well as time to nearest high tide for both study sites at (b) night and (d) day, respectively, according to final model in Table 2. Bold and dotted lines: model predictions; shaded areas: 95% credible intervals

The trip distances of oystercatchers differed between the two study sites, according to both time of day and time to nearest high tide (significant interactions in Table 2; according parameter estimates are shown in Table 3). However, trip distances increased strongly with increasing distance between the nest and high‐tide line consistently at both study sites (Figure 3; Table 2). This relationship was true for trips during daytime (Figure 3a) and nighttime (Figure 3c). Oystercatchers breeding further from the high‐tide line thus searched for food on more distant tidal flats than oystercatchers breeding closer to the high‐tide line. Foraging trip distances only increased significantly with increasing time to next high tide for oystercatchers on Oland, with the difference between the two colonies becoming significant at a time of >260 min to the next high tide (Figure 3). Furthermore, differences in foraging trip distances between the two study sites were greater at night (Figure 3d) than during the day (Figure 3b). There were no differences in foraging trip distances between night and day within the same study site, and no significant differences in trip distances among the study years (Table 2).

**Table 2:** Results of likelihood ratio tests of main effects and interaction terms with respect to the dependent variables maximum trip distance to nest and trip duration (log‐transformed). *n* = 548 trips in 29 individuals; rm = nonsignificant interaction term removed from the model. Interaction terms not significant for either trip distance or trip duration are not shown. Interactions were removed from the model for testing the main effects

	Max. trip distance to nest			Trip duration		
	*df*	LR	*p*	*df*	LR	*p*
Year	2	3.67	0.16	2	4.47	0.107
Colony	1	3.2	0.074	1	3.23	0.072
Time of day	1	1.45	0.228	1	103.98	**<0.001**
Time till high tide	1	79.438	**<0.001**	1	0.15	0.7
Nest distance	1	22.85	**<0.001**	1	3.55	0.05
Colony × Time till high tide	1	63.472	**<0.001**	1	10.02	**<0.001**
Colony × Time of day	1	4.01	**0.042**			rm

*df*, degrees of freedom; LR, results of likelihood ratio test.

**Table 3:** Parameter estimates of the final model including 95% credible intervals (CrI) in brackets

	Max. trip distance to nest estimate (95% CrI)	Trip duration estimate (95% CrI)
Intercept	6.27 (5.75 to 6.76)	3.04 (2.62 to 3.46)
Year 2009	0.33 (−0.40 to 1.06)	0.35 (−0.23 to 0.93)
Year 2010	0.92 (−0.12 to 1.97)	0.80 (−0.20 to −1.62)
Colony (Spiekeroog)	−0.51 (−1.30 to 0.25)	−0.47 (−1.0 to −0.13)
Time of day (night)	0.30 (0.05 to 0.25)	0.99 (0.80 to 1.17)
Time to high tide	0.62 (0.52 to 0.71)	0.20 (0.06 to 0.34)
Nest distance	0.65 (0.40 to 0.91)	0.16 (−0.04 to 0.36)
Colony (Spiekeroog) × time to high tide	−0.49 (−0.61 to −0.37)	−0.26 (−0.42 to −0.10)
Colony (Spiekeroog) × time of day (night)	−0.30 (−0.59 to 0.00)	rm

**Figure 4:**
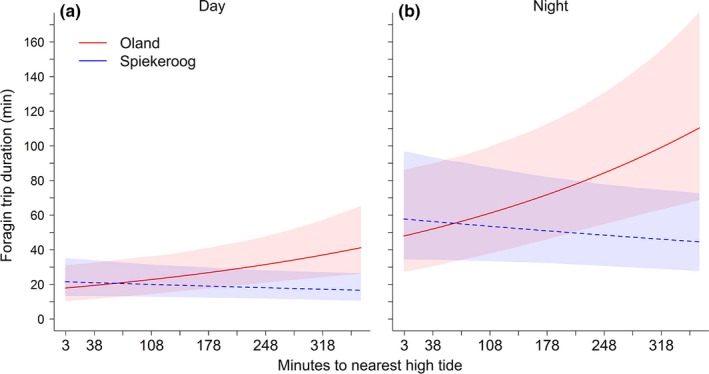
Model predictions for foraging trip durations during (a) day and (b) night according to the final model in Table 2. Bold and dotted lines: model predictions; shaded areas: 95% credible intervals

Trip durations differed between the two study sites, according to time to nearest high tide (significant interaction in Table 2). In contrast to foraging trip distance, there was no significant effect of distance between nest and high‐tide line on trip duration (Table 2). However, time to nearest high tide had a significant effect on the duration of foraging trips; foraging trips on Oland always lasted longer with increasing time to the nearest high tide, while this correlation was not evident on Spiekeroog (Figure 4). Foraging trips took significantly longer at night than during the day at both study sites (Table 2; Figure 4). Night foraging trips for oystercatchers breeding on Oland were significantly longer than those for oystercatchers breeding on Spiekeroog, with the differences mainly attributable to foraging trips performed during low tide (Figure 4). There were no significant differences in foraging trip duration among the study years (Table 2).

## 4 Discussion

### 4.1 Effect of nest distance

Oystercatchers consistently travelled significantly longer distances to forage with increasing distance between their nest site and the high‐tide line, thereby supporting our first hypothesis. This pattern was true for both study sites, located on different types of islands in different parts of the Wadden Sea, and over a period of 3 years, suggesting that it is likely to be a general feature. These results support the idea of a steady increase in breeding‐site quality with decreasing distance from the high‐tide line, given that individual oystercatchers breeding closer to the high‐tide line can reduce their foraging trip distances across the tidal flats. These results provide quantitative support for the concept presented by Ens et al. (1992).

In contrast, we found no significant effect of breeding‐territory location on foraging trip duration, rejecting our second hypothesis. Although leapfrog oystercatchers obviously need to invest more time in flight than residents (compare Ens et al., 1992; Kersten, 1996; this study) by traveling further from their nests to the high‐tide line, as well as traveling further from the high‐tide line to their more distant foraging territories, our results showed that leapfrog oystercatchers spent similar lengths of time on foraging trips as residents. This suggests that oystercatchers occupying low‐quality breeding territories may try to minimize the time spent in their foraging territories. Swennen, Leopold, and Bruijn (1989) showed that time‐limited oystercatchers were able to increase their food intake rate by decreasing their searching and handling times, which could explain why trip durations were similar irrespective of the distances between the nest site and the high‐tide line. Besides altering their foraging behavior, oystercatchers may compensate for limited time by shortening times of preening, resting, or social interactions (Swennen et al., 1989) which may further lead to disadvantages for leapfrog oystercatchers. It is very likely that even in leapfrog oystercatchers the commuting time between the breeding and the foraging territory is only a minor part of the overall time spent away from the nest site. This means that foraging trip duration was much more influenced by the prey base (intake rates) as well as other behaviors such as resting or preening. It would have extended the scope of the current study (and would have been impossible during nighttime anyway) to record intake rates in different parts of the intertidal flats. However, the prey base around both study sites had been investigated already in detail (Schwemmer et al., 2016), and those data generally showed high prey abundance and biomasses in areas further down the shore which were often frequented by leapfrog oystercatchers. Therefore, the missing significant relationship between foraging trip duration and distance from nest to the high‐tide line may be explained by differences in the prey base rather than a longer time needed to commute between breeding and foraging territories.

Furthermore, when drawing conclusions about time budgets, it is important to consider that our study was only performed during the incubation period, and time budgets are likely to differ during chick rearing, as shown in earlier studies (Kersten, 1996).

Finally, due to the close vicinity of breeding and feeding territories in resident birds, several trips might not have been identified. However, the spatial resolution of the GPS devices is very precise (generally more accurate than 20 m). Thus, we are confident that GPS fixes from breeding territories could be well separated from those within feeding territories. Moreover, the log interval of 2 min should provide a high enough temporal resolution as visual observations of resident birds never showed trip durations of <2 min (Schwemmer & Garthe, 2011).

### 4.2 Effect of tide

In accordance with our third hypothesis, tidal stage affected both the distance and duration of foraging trips. However, in contrast to the relationships between foraging trip distance and distance between the nest and high tideline, we found greater differences between the two study sites, suggesting that the effect of tidal stage on foraging trip characteristics was site‐dependent. This was most likely caused by site‐specific differences in tidal conditions such as higher lying intertidal flats surrounding Oland as compared to Spiekeroog. Oystercatchers breeding on Oland followed the tide line, given that both foraging trip distance and duration increased with increasing time to the next high tide. In contrast, tidal stage had little effect on foraging trip characteristics for oystercatchers breeding on Spiekeroog. Besides different inundation times, these site‐specific patterns might be related to differences in prey bases during the tidal cycle (e.g., de Vlas, Bunskoeke, Ens, & Hulscher, 1996) or different prey availabilities between the two study sites (Schwemmer et al., 2016). During the study period, oystercatchers on Spiekeroog intensively used cockles (*Cerastoderma edule*, L.) as their main prey, while oystercatchers on Oland additionally used American razor clams (*Ensis directus*, conrad) (Schwemmer et al., 2016), which typically occur in low‐lying intertidal flats distant from the breeding sites (Armonies & Reise, 1999). Visiting low‐lying, remote intertidal flats may thus explain the large increase in foraging trip distance and duration with increasing time to next high tide observed on Oland, given that the razor clam fields were only exposed at extreme low tide.

Because the effect of tidal stage appears to be site‐specific, our findings suggest that the location of the breeding territory relative to the high‐tide line is the most crucial factor determining foraging trip distances of oystercatchers.

### 4.3 Effect of time of day

Finally, we found clear evidence from both study sites to support our fourth hypothesis, that is, that foraging trips would take significantly longer during the hours of darkness, compared with trips during daylight hours (in contrast, time of day had no effect on foraging trip distance), in accordance with earlier work by Schwemmer and Garthe (2011). There are two possible explanations for this. Firstly, some previous studies suggested that oystercatchers might spend more time foraging at night because they need to forage using tactile, rather than visual cues (e.g., Sitters, 2000; Sutherland, 1982). However, other studies found no difference in food‐consumption rates between day and night in either captive (Hulscher, 1996) or free‐living oystercatchers (Kersten & Visser, 1996). Alternatively, Leopold, van Elk, and van Heezik (1996) pointed out that oystercatchers should minimize the time spent away from the breeding site to reduce predation pressures on eggs and chicks. However, neither of the study sites had any nocturnal mammalian predators, and the main predation pressure was from gulls during daylight hours (K. Lutz & M. Scheffler, unpublished; our own observations). This may therefore explain why oystercatchers extended their nighttime foraging trip durations, while maximizing the time spent within their breeding territory during the day, to prevent predation by diurnal avian predators.

Swennen et al. (1989) pointed out that oystercatchers acting as tactile feeders during the night may have difficulties to capture *Ensis* because this shellfish has the ability to rapidly retreat down its burrow, out of the reach of the oystercatcher′s bill. Our GPS data, however, suggest that oystercatchers from Oland visited the *Ensis* sand flat during the night as often as they did during daytime. As no other potential prey species were available on this particular sand flat (Schwemmer et al., 2016) and we observed oystercatchers feeding on individuals of *Ensis* which were sticking out of the surface and being hardly alive, it is very likely that oystercatchers were able to feed on this prey during the night using tactile means.

## 5 Conclusions

Overall, the results of this study support and quantify the concept presented by Ens et al. (1992), by detecting a linear relationship between nest‐site location and foraging trip distance. Ens et al. (1992) separated breeding territories into two classes based on quality: low quality (leapfrogs) and high quality (residents). However, our results suggest that nest‐site quality might decrease steadily with increasing distance from the high‐tide line. This is in line with observations made by Heg et al. (2000) who stated that leapfrogs with breeding territories adjacent to residents were more likely to acquire a resident territory in the future. Further studies are needed to record more parameters describing territory quality, such as fledging success, food intake rate, and activity budgets, and to relate them to distance between the breeding territory and the high‐tide line.

GPS data loggers allowed us to record even very distant foraging trips, as well as trips performed at night, neither of which could be recorded visually, given that the longest oystercatcher foraging trip was almost 6 km from the high tideline and thus out of range of telescopes. These long distances are in contrast to the observations of Ens et al. (1992), who reported trip distances of 200–500 m in leapfrog oystercatchers.

Although our data clearly support Ens et al.'s (1992) concept that leapfrog oystercatchers need to travel further to their foraging sites than residents, the concept of the birds having relatively static foraging territories on the intertidal mudflats (Figure 1) is less certain, even though it has already been adopted in a series of follow‐up studies (e.g., Hulsman, Zalucki, & Iedema, 1996; Heg et al., 2000; Bruinzeel et al., 2006; but see Kersten & Visser, 1996).

**Figure 5:**
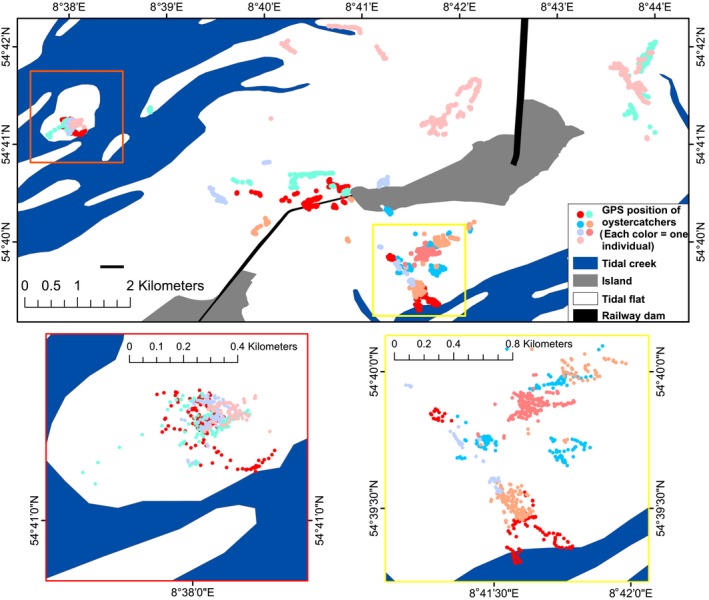
Distribution of seven equipped oystercatchers in their feeding territories around Hallig Oland during 2009. Each dot represents a GPS fix, each color an individual oystercatcher. The yellow‐framed right inset zooms into an area closer to the island, where the overlap between foraging territories is moderate to low. The red‐framed left inset zooms into an area further away from the island (low‐lying razor clam field), where overlap between foraging territories of oystercatchers is intense

Indeed, we found evidence for much less static feeding habitats further away from the shore: Closer to the island (and closer to the high‐tide line) oystercatchers tended to overlap only very little with conspecifics, although they did not use single feeding territories but rather switched between different sites (yellow box and right inset in Figure 5). In contrast, further away from the breeding territories (and closer to the low‐tide line) GPS fixes of different oystercatchers overlapped strongly (red box and left inset in Figure 5). This high degree of overlap, indicating a rather nonstatic nature of feeding habitats at least far away from the breeding territories might have two reasons: (1) As already found by Ens et al. (1992), using visual cues, especially territories of leapfrog oystercatchers may vary as conspecifics might use the vacated feeding territory when leapfrogs are absent to attend their breeding territories. (2) The high abundance of razor clams in this area (Schwemmer et al., 2016) might have facilitated high intake rates leading to a lower degree of competition among individual oystercatchers.

It cannot be ruled out that the differences in foraging trips between the study of Ens et al. (1992) and this study might have been also caused by differences in study location. However, as we have observed consistent patterns in two different sites, it is likely that the observed patterns are a general feature. Furthermore, trip patterns of oystercatchers in our study might differ from the ones reported by Ens et al. (1992) because we were only able to collect data during the incubation period. It may well be that foraging trips are different during the chick‐rearing phase, when adults need to feed their chicks on a constant basis.

In agreement with Ens et al. (1992), our data generally supported the idea that oystercatchers occupying a breeding territory far from the high‐tide line need to forage in more distant areas than individuals breeding close to the high‐tide line. The data presented in this study extent this concept by showing that there is a steady decrease in nest‐site quality with increasing distance from the high‐tide line.

## Conflict of Interest

The authors state that they have no conflict of interest.

## Data Accessibility

Bird‐ringing data are archived at the Beringungszentrale Vogelwarte Helgoland (Institute for Avian Research, Wilhelmshaven). Raw tracking data are stored at www.movebank.org.
